# Loneliness and fearfulness are associated with non-fatal drug overdose among people who inject drugs

**DOI:** 10.1371/journal.pone.0297209

**Published:** 2024-02-21

**Authors:** Oluwaseun Falade-Nwulia, Kathleen Ward, Karla D. Wagner, Hamidreza Karimi-Sari, Jeffrey Hsu, Mark Sulkowski, Carl Latkin, Evaristus Nwulia

**Affiliations:** 1 Division of Infectious Diseases, Johns Hopkins University School of Medicine, Baltimore, MD, United States of America; 2 School of Public Health, Drexel University, Philadelphia, PA, United States of America; 3 School of Public Health, University of Nevada, Reno, NV, United States of America; 4 Department of Psychiatry and Behavioral Science, Johns Hopkins University School of Medicine, Baltimore, MD, United States of America; 5 Department of Psychiatry, Howard University, Washington, DC, United States of America; University of Washington, UNITED STATES

## Abstract

**Background:**

People who inject drugs (PWID) experience high rates of drug overdose death with the risk of mortality increasing after each non-fatal event. Racial differences exist in drug overdose rates, with higher rates among Black people who use drugs. Psychological factors may predict drug overdose.

**Methods:**

Cross-sectional data from a survey administered to PWID in Baltimore, MD enrolled in a social network-based intervention were analyzed. Linear regression methods with generalized estimating equations were used to analyze data from indexes and network members to assess for psychological factors significantly associated with self-reported number of lifetime drug overdoses. Factors associated with number of overdoses were assessed separately by race.

**Results:**

Among 111 PWID enrolled between January 2018 and January 2019, 25.2% were female, 65.7% were Black, 98.2% reported use of substances in addition to opioids, and the mean age was 49.0 ± 8.3 years. Seventy-five individuals (67.6%) had a history of any overdose with a mean of 5.0 ± 9.7 lifetime overdoses reported. Reports of feeling fearful (β = 9.74, P = 0.001) or feeling lonely all of the time (β = 5.62, P = 0.033) were independently associated with number of drug overdoses. In analyses disaggregated by race, only the most severe degree of fearfulness or loneliness was associated with overdose among Black participants, whereas among White participants, any degree of fearfulness or loneliness was associated with overdose.

**Conclusions:**

In this study of PWID loneliness and fearfulness were significantly related to the number of reported overdose events. These factors could be targeted in future interventions.

## 1. Introduction

Over the last two decades, fatal and non-fatal drug overdose rates have continued to climb in the United States [1,2]. PWID who experience a non-fatal overdose are at an increased risk of subsequent fatal overdose, with about 1 in 5 PWID at an elevated risk of fatal overdose each year with a dose-dependent relationship between number of non-fatal overdoses and overdose death [3,4]. Current evidence-based interventions for overdose prevention include medication for opioid use disorder [5], access to naloxone [6,7], and fentanyl testing strips [8]. However, racial disparities in access to such services exist, and challenges remain with identifying patients at the highest risk of overdose mortality and thus in need of increased support to access these lifesaving interventions as well as more wrap-around services [9,10]. In addition, these interventions do not address the root causes or psychological consequences of unintentional drug overdose and are slow to adapt to an ever-evolving toxic drug supply such as the introduction of xylazine which is unresponsive to naloxone [11]. As more PWID experience multiple overdose events in their lifetime due to the contaminated drug supply [12], an in-depth understanding of a variety of predictors of risk, such as psychological factors, are needed to guide development of more targeted interventions that attend to and ameliorate overdose fatality.

The self-medication theory of substance use suggests that many people with hazardous levels of substance use do so as a mechanism to cope with underlying distress [13] More recently, research has examined mental health-related predictors of overdose. In one study among overdose survivors, depression, anxiety, and substance use disorder diagnoses were associated with a higher hazard of repeated overdose [14]. In a population-based study of 272,561 US adults, psychological distress was found to be associated with risk of overdose death with an over 10-fold increase in hazards of overdose death among individuals endorsing high psychological distress compared to those with no psychological distress [15]. Discrimination, racial isolation, adversity due to trauma, and psychosocial stressors such as low socio-economic status and income inequality, which are more prevalent among Black populations compared to White populations may predispose Black individuals to increased psychological distress and increased vulnerability to maladaptive coping strategies, including increased substance use [16].

The United States experienced an approximate 30% increase in drug overdose death from 2019–2020 with 91, 799 overdose deaths reported in 2020 [1]. The age-adjusted fatal overdose rate increased an additional 14% in the next year with a total of 106, 699 overdose deaths reported in 2021. Racial disparities in drug overdose deaths also increased. An analysis of surveillance data from 26 US jurisdictions found that Black Americans experienced the highest relative rate increase in drug overdose death rates (44%, compared to 22% among White Americans during 2019–2020 [17], a period coinciding with high psychological distress, loneliness and social isolation due to the COVID-19 pandemic mandated social distancing measures.

The relationship between social interactions and substance use has been demonstrated in animal studies, showing that socially isolated (i.e., lonely) animals used more drugs than socially enriched animals [18]. Mechanistically, social deprivation negatively impacted structure and function of brain regions involved in decision making and response to drug rewards [19]. Exploration of the role of loneliness in substance use and on substance use outcomes is nascent [20]. This analysis aims to understand psychological factors associated with experiences of drug overdose and their differential impact by race. We hypothesized that increased loneliness would be associated with increased lifetime overdose experiences among a cohort of PWID and that the effect would differ for Black and White PWID.

## 2. Methods

This study utilized data from the social network-based intervention study CHAMPS CONNECT, details of which have been described elsewhere [21]. Briefly, social networks of PWID were recruited from one infectious disease clinic and community-based sites in Baltimore, MD between 1/25/2018 and 1/4/2019. Data were derived from an interviewer-administered survey completed by all enrolled participants. Index participants were 18 years or older, English speaking, hepatitis C virus (HCV) antibody positive and reported injection drug use with another person in the past year. Indexes were also asked to complete a network inventory which elicited names of their network members, including drug network members. These indexes were then asked to recruit their injection drug network members for HCV testing and linkage to care. Consistent with other network studies, indexes received $10 for each network member enrolled into the study and $50 for completing the survey and network inventory. The Johns Hopkins University School of Medicine Institutional Review Board approved the study protocol. Each participant signed a written informed consent document before enrollment into this study.

### 2.1.Study measures

The primary outcome, number of overdoses, was assessed based on the response to the question, “how many times in your life have you overdosed?”. Questions from the Center of Epidemiologic Studies Depression Scale (CES-D) [22] were used to assess for the association between psychological factors and experiences of drug overdose. Questions probed on psychological symptoms in the past 7 days and responses were categorized as 1) little of the time for responses of “rarely or none of the time (less than one day)”, 2) occasionally for responses of “some or a little of the time (1–2 days)” and 3) all of the time for responses of “a moderate amount of time (3–4 days)”, and “all of the time (5–7 days)".

The Alcohol Use Disorders Identification Test-Consumption (AUDIT-C) was used to assess for risky alcohol use [23]. Other variables including education, employment, marital status, having children, homelessness, health insurance, monthly income (US$), history of incarceration, lifetime substance use, frequency of injection during the past 30 days, history of drug treatment, and history of psychiatric treatment were assessed based on responses to the interviewer administered survey.

### 2.2.Statistical analyses

All statistical analyses were conducted using Stata 16 software (College Station, TX) [24]. Summary statistics for patterns of distribution of categorical (e.g., racial/ethnic groups, gender, homelessness, and lifetime psychiatric diagnosis) and continuous (current age, monthly income, and age at first drug use) variables, were computed for two groups of participants with no lifetime overdose versus one or more lifetime overdoses. P-values for inferences on the differential distributions of these characteristics between the two groups (i.e., lifetime overdose as dichotomous outcome) were derived from simple (i.e., univariate) logistic regressions with generalized estimating equations, which accounts for clustering within network members. We used a linear regression approach to examine the primary aims of the study, i.e., predictors of non-fatal overdoses as a continuous outcome. Multiple linear regression with generalized estimating equations were used to determine factors independently associated with number of lifetime drug overdoses, and regression coefficient (β), standard error (SE), and significance (P) were reported. Factors including gender, race, homelessness, employment status, having children, educational attainment, history of incarceration, and income previously known or hypothesized to be associated with overdose were included in the adjusted analyses [25,26]. Marginal plots were run post regression analyses to highlight predicted mean and 95% confidence interval (CI) of number of lifetime overdoses for three race categories (Black, White, Other) conditional on the severity of self-reported fearfulness and loneliness. Factors that demonstrated an association at the P < 0.05 level were considered statistically significant.

## 3. Results

Among 111 PWID enrolled, 28 (25.2%) were female, 73 (65.7%) were Black, 109 (98.2%) reported use of other substances in addition to opioids, and the mean age (± SD) of the sample was 49.0 ± 8.3 years. Depressive symptoms were common, with a mean score of 22.0 (± 5.6) on the CES-D 10 (Table 1). Seventy-five individuals (67.6%) had a history of overdose with a mean number (± SD) of 5.0 ± 9.7 lifetime overdoses. Compared to individuals without overdose history, those with an overdose history were younger at injecting drug use initiation (22.3 ± 7.7 vs. 25.9 ± 10.5 years, P = 0.036). Across both groups, the majority were unemployed (86.4%), had health insurance ((96.4%), had previously been incarcerated (96.4%), and had previously been in drug treatment (96.4%) with no significant differences between individuals who had experienced lifetime overdose and those who had not (p>0.05). There was a trend towards a lower proportion of women in the lifetime overdose history group compared to the no lifetime overdose history group (20.0% vs. 36.1%, P = 0.068).

**Table 1:** Demographic, social, and behavioral characteristics by lifetime overdose history in a sample of PWID enrolled in a social network-based HCV intervention in Baltimore, Maryland*, **.

Characteristics	Total (N = 111)	Lifetime overdose history		*P* value
		Yes (N = 75)	No (N = 36)	
**Overdoses number, n**	-	5.0 ± 9.7	-	-
**Age, years**	49.0 ± 10.4	48.5 ± 11.0	50.0 ± 8.9	0.447
**Female**	28 (25.2%)	15 (20.0%)	13 (36.1%)	0.068
**Race/ethnicity**				
** Black/African American**	73 (65.8%)	45 (60.0%)	28 (77.8%)	Reference
** White**	32 (28.8%)	25 (33.3%)	7 (19.4%)	0.252
** Other**	6 (5.4%)	5 (6.7%)	1 (2.8%)	0.241
**Education**				
<12^th^ grade	40 (36.0%)	29 (38.7%)	11 (30.6%)	Reference
** High school/GED**	43 (38.7%)	29 (38.7%)	14 (38.9%)	0.662
** College**	28 (25.2%)	17 (22.7%)	11 (30.6%)	0.197
**Unemployed**	96 (86.5%)	67 (89.3%)	29 (80.6%)	0.205
**Married**	13 (11.7%)	9 (12.0%)	4 (11.1%)	0.892
**Have children**	75 (67.6%)	52 (69.3%)	23 (63.9%)	0.571
**Homeless**	54 (48.7%)	39 (52.0%)	15 (41.7%)	0.308
**Health insurance**	107 (96.4%)	73 (97.3%)	34 (94.4%)	0.447
**Monthly income, US$**	1004.8 ± 1245.3	1048.6 ± 1417.7	913.6 ± 781.9	0.520
**History of Incarceration**	107 (96.4%)	72 (96.0%)	35 (97.2%)	0.752
**Lifetime duration of incarceration, months**	101.1 ± 124.6	109.5 ± 128.5	83.6 ± 115.9	0.292
**AUDIT score**	3.19 ± 3.63	2.92 ± 3.64	3.75 ± 3.56	0.258
**CES-D-10 score**	22.0 ± 5.6	22.1 ± 5.9	21.7 ± 5.1	0.706
Age at 1^st^ IDU, years	**23.5 ± 8.8**	**22.3 ± 7.7**	**25.9 ± 10.5**	**0.036**
**Frequency of injection in past 30 days**				
** None**	25 (22.5%)	20 (20.7%)	5 (13.9%)	Reference
** 1–6 days per week**	39 (35.1%)	24 (32.0%)	15 (41.7%)	0.112
** Daily**	47 (42.3%)	31 (41.3%)	16 (44.4%)	0.210
**Drug treatment**				
** Lifetime**	107 (96.4%)	73 (97.3%)	34 (94.4%)	0.594
** Past 6 months**	**71 (64.0%)**	**53 (70.7%)**	**18 (50.0%)**	**0.034**
**Lifetime psychiatric diagnosis**	67 (60.4%)	46 (61.3%)	21 (58.3%)	0.762
**Psychiatric treatment in past 6 months**	**56 (50.5%)**	**43 (57.3%)**	**13 (36.1%)**	**0.036**

* Data are presented as numbers (%) and mean ± standard deviation.

** Abbreviations: Alcohol use disorders identification test (AUDIT); Center for Epidemiologic Studies depression scale (CES-D); injection drug use (IDU).

Compared to those without a lifetime history of overdose, those with a lifetime history of overdose were more likely to have received drug treatment (70.7% vs. 50.0%, P = 0.034), and psychiatric treatment (57.3% vs. 36.1%, P = 0.036) within the past six months.

In simple regression analyses, self-reported lifetime uses of methamphetamine (β = 3.28, P = 0.05), barbiturates (β = 3.88, P = 0.02) and inhalants (β = 4.26, P = 0.01) were either modestly or significantly associated with number of drug overdose events but were no longer associated after controlling for gender, race, homelessness, employment status, having children, educational attainment, average income over six months, and total duration of lifetime spent incarcerated (Table 2). Opioids were not included in regression analyses as all participants reported lifetime use of opioids.

**Table 2:** Simple and multiple regression analysis of total lifetime number of drug overdoses on self-reported lifetime substance use among a sample of PWID enrolled in a social network-based HCV intervention study in Baltimore, MD*.

Substance use variables (self-reported lifetime use)	Total (N = 111)	Total lifetime drug overdose events					
		Unadjusted association			Adjusted association **		
		β	SE	*P*	β	SE	*P*
**Methamphetamine**	33 (29.7%)	3.28	1.69	0.052	1.59	2.01	0.430
**Barbiturates**	35 (31.5%)	**3.88**	**1.64**	**0.018**	2.72	1.74	0.118
**Benzodiazepine**	56 (50.5%)	1.82	1.55	0.243	1.18	1.59	0.459
**Cocaine powder**	102 (91.9%)	2.44	2.85	0.391	2.14	2.88	0.457
**Crack cocaine**	84 (75.7%)	0.69	1.84	0.706	0.15	1.88	0.937
**Speedball**	104 (93.7%)	1.97	3.22	0.541	0.70	3.13	0.824
**Inhalants**	29 (26.1%)	**4.26**	**1.73**	**0.014**	3.44	1.84	0.062
**Hallucinogens**	45 (40.5%)	2.18	1.58	0.168	0.07	1.92	0.971

* Data are presented as the regression coefficient (β), standard error (SE), and significance (*P*).

** Multiple linear regression of drug use characteristics adjusted for gender, race/ethnicity, homelessness, employment status, having children, educational attainment, average income over 6 months and total duration of lifetime incarceration.

Reports of feeling depressed all of the time (β = 4.77, P = 0.06), fearful all of the time (β = 9.52, P <0.01), or lonely all of the time (β = 6.07, P = 0.02) were either modestly or significantly associated with number of drug overdose events. Reports of feeling fearful all of the time (β = 9.74, P = 0.001) or feeling lonely all of the time in the past 7 days (β = 5.62, P = 0.033) were independently associated with number of lifetime drug overdoses after adjusting for gender, race, homelessness, employment status, having children, educational attainment, average income over six months and total duration of lifetime incarceration (Table 3). The association between severity of fearfulness and drug overdoses remained significant after correcting for multiple testing (Bonferroni-corrected P value set at 0.005).

**Table 3:** Simple and multiple linear regression analyses of total lifetime number of drug overdoses on lifetime psychiatric diagnosis, CES-D-10 questionnaire items, and total CES-D-10 score in a sample of PWID enrolled in a social network-based HCV intervention in Baltimore, MD.

Psychological variables	Total (N = 111)	Total lifetime drug overdose events					
		Unadjusted association			Adjusted association*		
		β	SE	*P*	β	SE	*P*
**CES-D: bother**							
** Little of the time**	43 (38.7%)	-0.62	1.78	0.726	0.35	1.77	0.844
** Occasionally**	40 (36.0%)	3.40	2.19	0.121	2.73	2.14	0.201
** All of the time**	28 (25.2%)	-0.25	3.12	0.936	-1.44	3.09	0.642
**CES-D: get-going**							
** Little of the time**	32 (28.8%)	1.74	1.90	0.358	2.17	1.85	0.241
** Occasionally**	44 (39.6%)	-0.51	2.06	0.802	-0.67	2.05	0.743
** All of the time**	35 (31.5%)	0.52	4.32	0.904	1.50	4.30	0.728
**CES-D: mind trouble**							
** Little of the time**	32 (28.8%)	-2.05	2.03	0.314	-2.96	2.01	0.141
** Occasionally**	32 (28.8%)	1.21	1.99	0.541	0.50	1.94	0.796
** All of the time**	47 (42.3%)	-0.71	2.75	0.797	-1.45	2.77	0.602
**CES-D: depressed**							
** Little of the time**	25 (22.5%)	-1.92	2.08	0.355	-2.47	2.13	0.246
** Occasionally**	35 (31.5%)	-0.69	2.07	0.740	-0.67	2.06	0.745
** All of the time**	51 (46.0%)	4.77	2.55	0.062	4.52	2.49	0.070
**CES-D: too much effort to do things**							
** Little of the time**	26 (23.4%)	-1.43	2.03	0.482	-0.61	2.12	0.773
** Occasionally**	41 (36.9%)	-1.69	2.22	0.447	-1.15	2.32	0.621
** All of the time**	44 (39.6%)	2.92	2.51	0.245	3.08	2.54	0.226
**CES-D: hopeless**							
** Little of the time**	27 (24.3%)	0.36	2.06	0.860	0.93	2.09	0.654
** Occasionally**	37 (33.3%)	3.17	2.28	0.164	4.09	2.27	0.071
** All of the time**	47 (42.3%)	0.86	2.31	0.711	1.79	2.32	0.440
**CES-D: fearful**							
** Little of the time**	48 (43.2%)	-1.08	1.70	0.523	-2.34	1.71	0.172
** Occasionally**	37 (33.3%)	0.41	2.14	0.848	-0.69	2.15	0.746
** All of the time**	**26 (23.4%)**	**9.52**	**3.07**	**0.002**	**9.74**	**2.93**	**0.001**
**CES-D: restless**							
** Little of the time**	28 (25.2%)	-0.12	2.20	0.955	0.79	2.34	0.734
** Occasionally**	27 (24.3%)	2.54	2.08	0.222	2.51	2.06	0.224
** All of the time**	56 (50.5%)	-0.27	2.32	0.907	-0.86	2.41	0.722
**CES-D: unhappy**							
** Little of the time**	16 (14.4%)	2.74	2.31	0.235	2.24	2.40	0.351
** Occasionally**	55 (49.6%)	0.57	2.52	0.820	-0.12	2.66	0.963
** All of the time**	40 (36.0%)	-0.07	3.28	0.982	-0.07	3.39	0.984
**CES-D: lonely**							
** Little of the time**	36 (32.4%)	-0.49	1.79	0.784	-0.75	1.83	0.680
** Occasionally**	46 (41.4%)	0.27	2.35	0.908	1.06	2.33	0.648
** All of the time**	**29 (26.1%)**	**6.07**	**2.65**	**0.022**	**5.62**	**2.64**	**0.033**
**Total CES-D score**		0.19	0.14	0.161	0.19	0.14	0.175

* Multiple linear regression of psychological factors adjusted for gender, race/ethnicity, homelessness, employment status, offspring, educational attainment, average income over 6 months, and total duration of lifetime spent in prisons.

**Fig 1:**
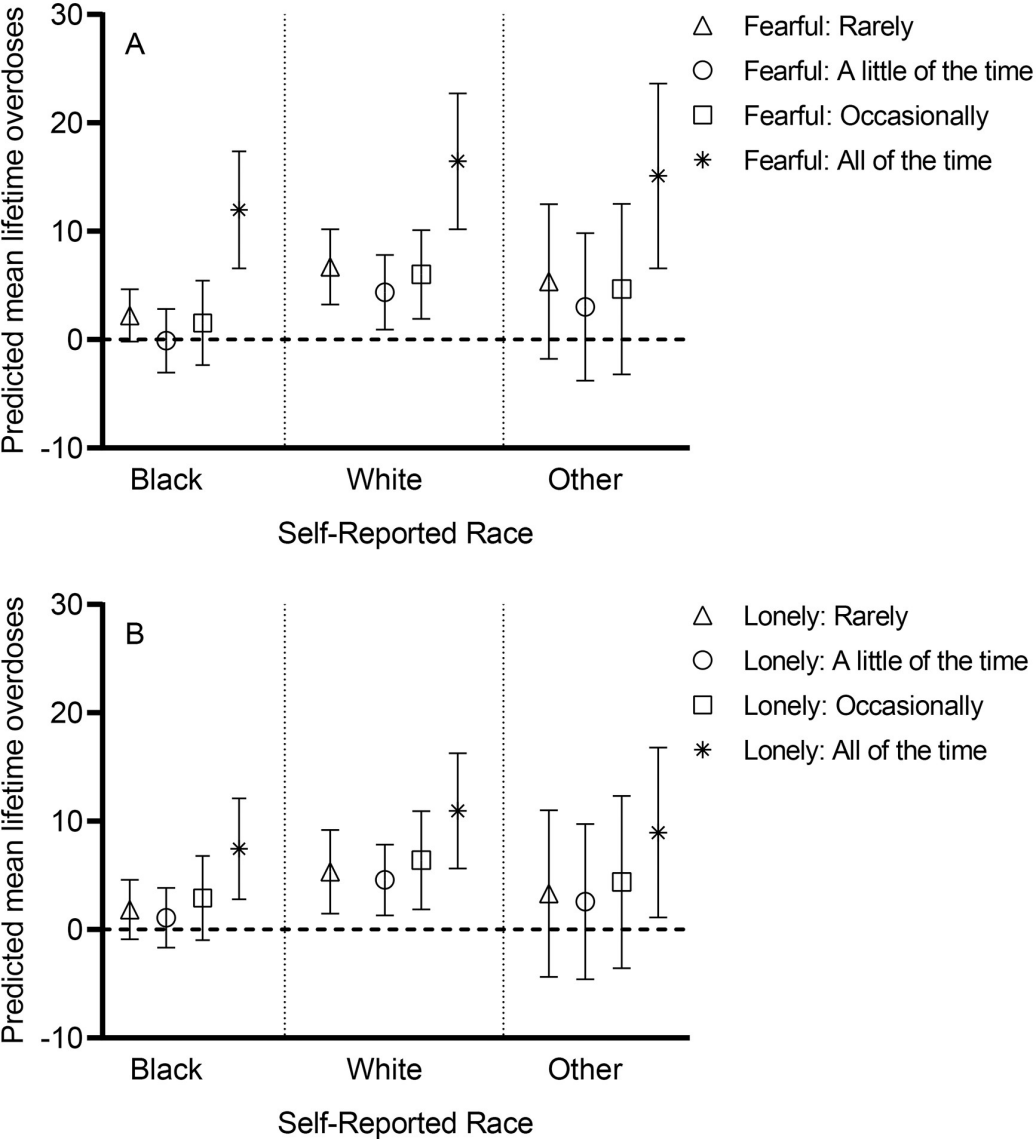
Predicted number of lifetime overdoses by race and severity of self-reported fearfulness and loneliness. Data expressed as mean and 95% confidence interval (CI).When a CI for a group includes zero mean (the horizontal dashed line), it implies that the predicted number of overdoses could also be zero for that group **(A)** Among Black participants (N = 73), the predicted mean number of overdoses was highest, and the 95% CI does not include zero for people fearful all of the time, compared to Black participants with lower degrees of fearfulness, where the 95% CI includes zero. A similar pattern is seen among those with race reported as other (N = 6). Among white participants (N = 32), predicted mean number of lifetime overdoses were significantly higher than zero for all degrees of fearfulness with the highest number of overdoses among those fearful all the time. **(B)** Among Black participants, the predicted mean number of overdoses was highest, and the 95% CI does not include zero for people lonely all of the time, compared to Black participants with lower degrees of loneliness, where the 95% CI included zero. A similar pattern is seen among those who report race as other. Among White participants, predicted mean lifetime overdoses were significantly higher than zero for all degrees of loneliness with the highest number of overdoses among those lonely all the time.

In an analysis of the association of fearfulness with overdose disaggregated by race, predicted number of overdoses was greatest and significantly different than zero for only the most severe degree of fearfulness (fearful all of the time) among Black participants (Fig 1A). Among White participants, predicted overdoses was significantly greater than zero irrespective of degree of fearfulness, with White participants who reported fearfulness all the time also experiencing the greatest number of non-fatal overdoses compared to white participants who rarely or occasionally experienced fearfulness. Similarly, in an analysis of the association of loneliness with overdose disaggregated by race, the predicted number of overdoses was highest and significantly different than zero only at the highest degree of loneliness (lonely all of the time) among Black participants (Fig 1B). Among White participants, predicted number of overdoses was significantly greater than zero irrespective of degree of loneliness with the highest number of predicted overdoses among White participants who reported loneliness all the time compared to White participants who experienced loneliness occasionally or a little of the time.

## 4. Discussion

This study identified fearfulness and loneliness as psychological items associated with lifetime number of drug overdoses. There are several mechanisms through which fearfulness and loneliness can be associated with overdose. Previous data suggest that PWID who witness an overdose are more likely to have an overdose event in the next six months [27]. Witnessing or experiencing an overdose may worsen feelings of both fearfulness and loneliness and has been associated with severe grief and loss responses [28–30]. Symptoms of fearfulness and loneliness are not specific to depressive disorders and may also be present in those with anxiety disorders, trauma history with post-traumatic stress disorder, and can be a symptom of worsening substance use disorder [31,32]. Conversely, fearfulness and loneliness may be symptoms of distress, and people who use drugs may be self-medicating through an increase in the amounts of substances used, predisposing them to overdose [31]. Additionally, an individual with symptoms of loneliness and fearfulness could start using more drugs, worsening social isolation and increasing the risk of fatal overdose. Overdose, loneliness, and fearfulness have complex bidirectional relationships that should be explored by future research.

Among Black PWID, only the most severe degrees of fearfulness and loneliness were significantly associated with the predicted number of overdoses. While the explanation for this is not immediately apparent, recent disparities in overdose outcome by race may warrant further evaluation of factors such as loneliness and fearfulness in overdose risk. Our findings of associations between lifetime history of overdose and current feelings of loneliness and fearfulness are particularly germane in the context of the current overdose crisis exacerbated by the COVID-19 pandemic. Quarantine and physical distancing policies implemented during the COVID-19 pandemic may have heightened feelings of loneliness and fearfulness among PWID potentially contributing to the increase in overdose numbers. Other data support the role of loneliness in exacerbating alcohol and other drug use during the COVID-19 pandemic [33–35].

Our findings suggest that fearfulness and loneliness may be specific factors associated with higher risk for drug overdose among PWID. This finding may warrant further screening/evaluation for loneliness and fearfulness with particular attention paid to interventions aimed at reducing anxiety and bolstering social supports. Peer-support interventions which have been shown to improve a wide range of outcomes for people who use drugs may be adapted to directly impact fearfulness and loneliness by creating additional social supports with an additional goal of reducing overdose risk. Recent data from a peer-facilitated substance use disorder care program implemented in an infectious disease care setting provides early data for the potential benefit of peer support on overdose risk [36]. Core components of the program included peer-based psychosocial support, provider training in SUD evaluation and care and prescription of medication for SUD treatment [37]. Participation in the program was associated with significant reduction in overdose risk at 3 months and 6 months compared to baseline [36,37]. Importantly, when asked to indicate helpful aspects of the program the majority (72%) selected “just knowing that someone cares” followed by phone calls from peers (54%) and the minority (32%) selected provision of medication for substance use disorder as helpful [37].

### 4.1.Limitations

Overdose was self-reported and may be affected by recall and reporting biases. Loneliness and fearfulness were also assessed using questions from the CES-D 10 which is validated as a 10-item measure of depression. The University of California-Los Angeles (UCLA) loneliness scale which is the most widely used measure of loneliness in populations of people with substance use [20] was not utilized in this study. The CES -D loneliness item used in this study has however been shown to be highly correlated with the 3-item UCLA loneliness scale and has been used as a measure of loneliness in other populations [38]. Due to the cross-sectional survey design, we could not assess the temporality of relationships. The sample size of 111 also limited our ability to detect significant associations in adjusted analyses. Additionally, this study was conducted among PWID enrolled in a hepatitis C intervention study majority of whom had a history of hepatitis C infection in an urban setting prior to the COVID-19 pandemic and may not reflect the experiences of people from rural settings or who use drugs but do not inject. Significant changes in psychological factors and responses to these factors may also have changed since the onset of the COVID-19 pandemic.

### 4.2.Conclusion

In our sample of predominantly Black PWID in Baltimore, Maryland, there was a high prevalence of lifetime overdose and the number of lifetime drug overdose events was significantly associated with current feelings of fearfulness and loneliness. Further work is needed to evaluate the role of loneliness and fearfulness in overdose risk in PWID communities.
